# Paranode Abnormalities and Oxidative Stress in Optic Nerve Vulnerable to Secondary Degeneration: Modulation by 670 nm Light Treatment

**DOI:** 10.1371/journal.pone.0066448

**Published:** 2013-06-19

**Authors:** Charis R. Szymanski, Wissam Chiha, Natalie Morellini, Nadia Cummins, Carole A. Bartlett, Ryan L. O'Hare Doig, Donna L. Savigni, Sophie C. Payne, Alan R. Harvey, Sarah A. Dunlop, Melinda Fitzgerald

**Affiliations:** 1 Experimental and Regenerative Neurosciences, School of Animal Biology, The University of Western Australia, Crawley, Western Australia, Australia; 2 Experimental and Regenerative Neurosciences, School of Anatomy, Physiology and Human Biology, The University of Western Australia, Crawley, Western Australia, Australia; Univeristy of Melbourne, Australia

## Abstract

Secondary degeneration of nerve tissue adjacent to a traumatic injury results in further loss of neurons, glia and function, *via* mechanisms that may involve oxidative stress. However, changes in indicators of oxidative stress have not yet been demonstrated in oligodendrocytes vulnerable to secondary degeneration *in vivo*. We show increases in the oxidative stress indicator carboxymethyl lysine at days 1 and 3 after injury in oligodendrocytes vulnerable to secondary degeneration. Dihydroethidium staining for superoxide is reduced, indicating endogenous control of this particular reactive species after injury. Concurrently, node of Ranvier/paranode complexes are altered, with significant lengthening of the paranodal gap and paranode as well as paranode disorganisation. Therapeutic administration of 670 nm light is thought to improve oxidative metabolism *via* mechanisms that may include increased activity of cytochrome *c* oxidase. Here, we show that light at 670 nm, delivered for 30 minutes per day, results in *in vivo* increases in cytochrome *c* oxidase activity co-localised with oligodendrocytes. Short term (1 day) 670 nm light treatment is associated with reductions in reactive species at the injury site. In optic nerve vulnerable to secondary degeneration superoxide in oligodendrocytes is reduced relative to handling controls, and is associated with reduced paranode abnormalities. Long term (3 month) administration of 670 nm light preserves retinal ganglion cells vulnerable to secondary degeneration and maintains visual function, as assessed by the optokinetic nystagmus visual reflex. Light at a wavelength of 670 nm may serve as a therapeutic intervention for treatment of secondary degeneration following neurotrauma.

## Introduction

Secondary degeneration of areas adjacent to an injury is a serious consequence of neurotrauma and results in additional loss of neurons, myelin and function [1], [2]. Neurons and glia vulnerable to secondary degeneration can undergo delayed death due to reactive metabolic events, *via* mechanisms thought to include Ca^2+^ overload, excess free radical formation and oxidative stress [3], [4]. Inflammation and death of cells often ensues [5]. Partial transection of the dorsal aspect of the optic nerve (ON) serves as a consistently reproducible model of secondary degeneration, allowing separation of the primary injury from ventral ON vulnerable to secondary degeneration [6], [7]. Using this model, we have demonstrated Ca^2+^ changes with early elevations in the oxidative stress indicators manganese superoxide dismutase (MnSOD) and carboxymethyl lysine (CML) in astrocytes vulnerable to secondary degeneration [8], [9], [10].

A significant feature of injury to the CNS is disruption and loss of myelin, with resultant functional impairment [11], [12]. In ON vulnerable to secondary degeneration, myelin decompaction is associated with long – term functional loss [13], [14]. Oligodendroglia are considered to be particularly vulnerable to excitotoxic insult and oxidative stress [15], although susceptibility varies depending upon maturation state [16]. Changes to ion channels and glutamate receptors have been demonstrated in various forms of white matter injury [17], [18]. Resultant disruption to the axoglial junction and node and paranode domains results in failure of saltatory conduction [19], [20], [21]. However, oxidative stress in oligodendrocytes and associated disruption of myelin specifically vulnerable to secondary degeneration has not been demonstrated *in vivo*.

Therapeutic administration of light in the red to far – red spectrum (630–1100 nm) alters the redox state of cytochrome *c* oxidase and is associated with activation of the enzyme, leading to effects consistent with increased flux through the electron transport chain and improvements in oxidative metabolism [22], [23]. Specifically, 670 nm light treatment increases mitochondrial membrane potential and reduces inflammatory mediators [24] and lipid peroxidation [25] in retina following light damage. As such, 670 nm light delivered by LED array is considered a safe, convenient and potentially effective antioxidant therapy in a range of preclinical models as well as clinical settings [26], [27], including during secondary degeneration [28]. Inhibition of cytochrome *c* oxidase activity was more detrimental to mature oligodendrocytes than oligodendrocyte precursor cells (OPCs) *in vitro* [29] but responses of oligodendroglia to increased cytochrome *c* oxidase activity are unknown. Here we demonstrate that alterations in indicators of oxidative stress in oligodendrocytes vulnerable to secondary degeneration are associated with abnormalities in node/paranode complexes. Short – term 670 nm light treatment reduces reactive species and limits paranode abnormalities. Furthermore, sustained long – term treatment is neuroprotective and preserves visual function.

## Results

### Oxidative stress indicators in oligodendrocytes vulnerable to secondary degeneration

**Figure 1:**
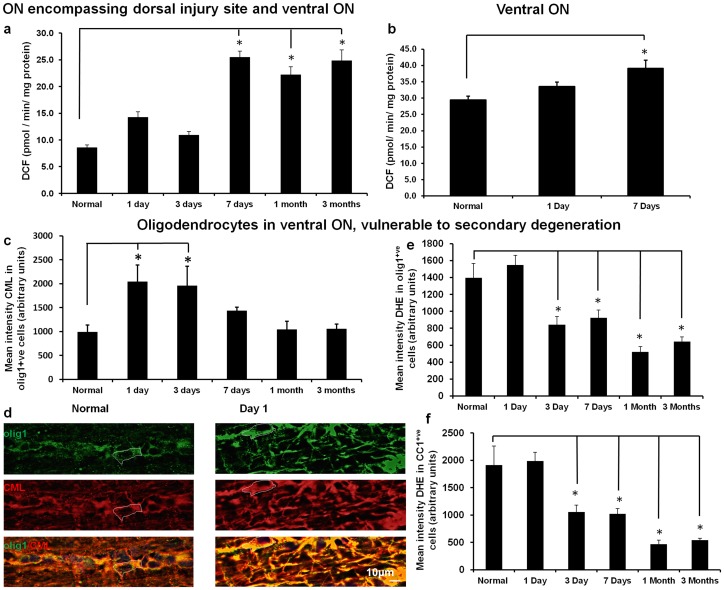
Oxidative stress indicators in ON after partial transection. (a) Mean ± SEM ROS/RNS assessed as DCF fluorescence in homogenates of ON including both the dorsal injury site and the ventral region vulnerable to secondary degeneration, from normal animals and 1, 3, 7 days, 1 and 3 months after injury, or (b) from ventral ON only from normal animals and 1 or 7 days after injury (6 animals pooled per time point, assayed in duplicate). (c) Semi – quantification of mean ± SEM intensity of CML immunoreactivity in olig1^+ve^ oligodendrocytes in ventral ON, assessed by tracing identified cells in single images in the z axis; representative images at 1 day (d), scale bar  = 10 μm, (n = 4–5 animals/time point). Similarly, semi – quantification of mean ± SEM intensity of DHE staining in olig1^+ve^ (e) or CC1^+ve^ (f) oligodendrocytes in ventral ON; * significantly different from normal (p≤0.05).

We first provided further evidence to indicate oxidative stress in ON following partial transection, and then looked more specifically to see if oligodendrocytes vulnerable to secondary degeneration were affected. There was a significant increase in ROS/RNS (as indicated by DCF fluorescence), in homogenates of the ON including both the dorsal injury site and the ventral region vulnerable to secondary degeneration, by 7 days after injury, with increases sustained at 1 and 3 months (Fig. 1a, p = 0.0033, dF = 5). DCF fluorescence in ventral ON homogenates was also significantly increased at day 7, compared to normal uninjured ventral ON (Fig. 1b, p = 0.029, dF = 5).

The oxidative stress indicator CML is an advanced glycation end – product of lipid peroxidation and glycoxidation reactions during oxidative stress [30]. CML immunointensity was assessed in oligodendrocytes of ventral ON vulnerable to secondary degeneration, using anti – olig1 to identify oligodendrocytes at varying degrees of maturity and anti – CC1 to identify mature oligodendrocytes [31], [32], [33]. CML was increased in ventral olig1^+ve^ oligodendrocytes at 1 and 3 days after injury (Fig. 1c, d, p = 0.014, dF = 5). The olig1^+ve^ oligodendrocytes assessed were relatively mature oligodendrocytes, as the olig1 immunoreactivity was cytoplasmic [31], [32] (Fig. 1d). This finding specifically indicates early oxidative stress in mature oligodendrocytes vulnerable to secondary degeneration. Furthermore, CML immunoreactivity in CC1^+ve^ mature oligodendrocytes [33] of ventral ON increased at day 1 after injury (normal  = 896.4±157.3; day 1  = 2685.6±380.5, p = 0.002, dF  = 3).

Oligodendrocytes of ventral ON immunopositive for olig1 displayed reduced intensity of DHE staining by 3 days after injury, which was sustained at 7 days, 1 and 3 months (Fig. 1e, p≤0.001, dF  = 5). Similar results were seen in mature CC1^+ve^ oligodendrocytes [33] (Fig. 1f, p≤0.001, dF  = 5). DHE has been used extensively as a superoxide specific marker [34], [35], [36], although detection of other ROS/RNS such as •OH, H_2_O_2_ and ONOO^−^ has been reported [37]. Nevertheless, our observed reduction in DHE staining in oligodendrocytes vulnerable to secondary degeneration is likely to reflect decreased ROS in the form of superoxide. We have previously reported increased MnSOD immunoreactivity in astrocytes in the first 3 days after injury [9] and, assuming superoxide detection by DHE in our system, our results indicate endogenous reduction of this particular ROS in ventral ON after partial transection. We observed no concurrent change in MnSOD immunoreactivity in ventral CC1^+ve^ oligodendrocytes (p>0.05, data not shown), indicating that reductions in superoxide *via* MnSOD may transcend cellular boundaries.

### Abnormal nodes of Ranvier and paranodes in ON vulnerable to secondary degeneration

**Figure 2:**
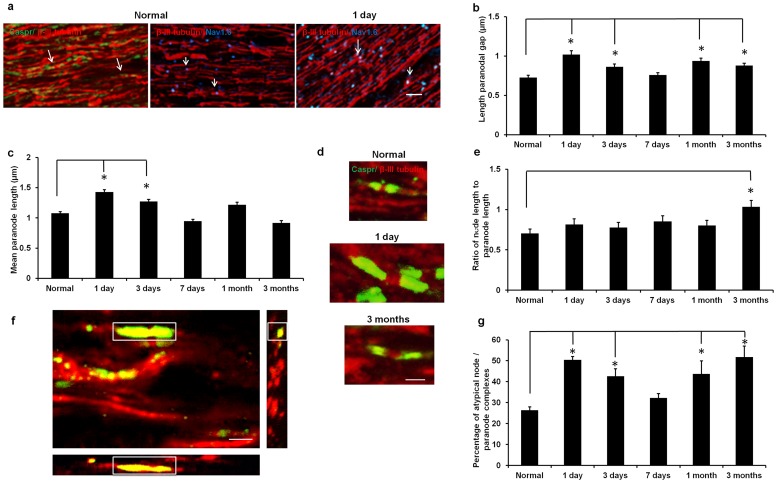
Node/paranode complexes in ventral ON after partial transection. (a) Representative images of Caspr^+ve^ paranodes (green) flanking the β-III tubulin^+ve^ (red) paranodal gap, and β-III tubulin^+ve^ areas (red) colocalised with Na_v_1.6^+ve^ nodes (blue) in normal ventral ON and at 1 day post injury; colocalised areas are yellow and purple respectively (examples indicated by arrows), scale  = 20 μm. Mean ± SEM length of the paranodal gap (b), paranode length (c) and the ratio of node to paranode lengths (e) in ventral ON of normal animals and 1, 3, 7 days, 1 and 3 months after injury; representative images (d), scale bar  = 1 μm. Orthogonal projection of a representative z stack illustrating a large atypical node/paranode complex well within the stack of images (f, boxed), scale bar  = 5 μm; Caspr^+ve^ paranodes are green, β-III tubulin^+ve^ axons are red (note: only projections in the z plane of the identified node/paranode complex are shown in the panels adjacent to the main image). Mean ± SEM percentages of node/paranode complexes that were atypical (hemi – nodes, single paranodes) (g), * significantly different from normal for each complex type (p≤0.05) (n = 6 animals/time point)

We have already established that structural abnormalities in the myelin sheath, such as decompaction, occur in ON vulnerable to secondary degeneration, but these changes are not observed until at least 3 months after injury [13], [14]. We wanted to know whether early oxidative stress in ventral oligodendrocytes was associated with earlier structural abnormalities that could occur as a consequence of dysfunctional oligodendrocytes. Consequently, we examined the structure of the axoglial junction, specifically the node/paranode complex. The paranodal gap, or space between the paranodes, was assumed to reflect the length of the node of Ranvier, as has been previously described [38]. The lengths of the paranodal gap and the average length of the two paranodes (Caspr^+ve^), in typical node/paranode complexes where nodes were flanked by two paranodes, were assessed in collapsed z stacks of ventral ON at increasing times after injury. The paranodal gap between Caspr^+ve^ paranodes was β-III tubulin^+ve^ and of similar size to Na_v_1.6^+ve^ areas on the axon (Fig. 2a). Both the paranodal gap and the average paranodal length were significantly increased at 1 and 3 days after injury, compared to normal (p = 0.019, dF  = 5, Fig. 2b – d). Increased lengths of the paranodal gap persisted at 1 and 3 months (p = 0.006, dF  = 5); the ratio of node length to paranode length was unchanged initially but was also significantly increased at 3 months (p = 0.004, dF  = 5, Fig. 2b, d, e). Lengthening of nodes (reflected by the paranodal gap) and paranodes are likely to reflect reduced concentration of ion channels at the node of Ranvier and disruption of the axoglial junction, with resultant reduced saltatory conduction [19], [20], supported by our reported loss of visual function following partial ON transection [8]. Immunointensity of Na_v_1.6 appeared slightly more diffuse at 1 day after injury (Fig. 2a), indicating reduced concentrations of ion channels, but the small scale of the observed differences precluded semi – quantification. The width of the paranode did not change in ON vulnerable to secondary degeneration (e.g. normal  = 0.50±0.01 µm, 1 day  = 0.56±0.02 µm, 3 months  = 0.48±0.01 µm; p>0.05).

Atypical node/paranode complexes (hemi – nodes or single paranodes) were identified in collapsed z stacks. We found that the percentages of atypical node/paranode complexes were significantly increased at 1 and 3 days and 1 and 3 months after injury (Fig. 2g, p = 0.014, dF  = 5), and were comprised of both hemi – nodes and single paranodes. Orthogonal projections were used to demonstrate that the majority of atypical complexes lay well within the captured images (example, Fig. 2f). Complete typical node/paranode complexes did not generally exceed more than 3 individual optical images in the z plane. As such, we are confident that missing Caspr^+ve^ paranodes are a reflection of altered immunoreactivity of paranodal material, perhaps reflecting structural abnormalities and/or disorganisation of the node/paranode complex, as discussed further below. Furthermore, the probability that part of the node/paranode complex was projecting out of the z stack is likely to be similar for all images assessed.

**Figure 3:**
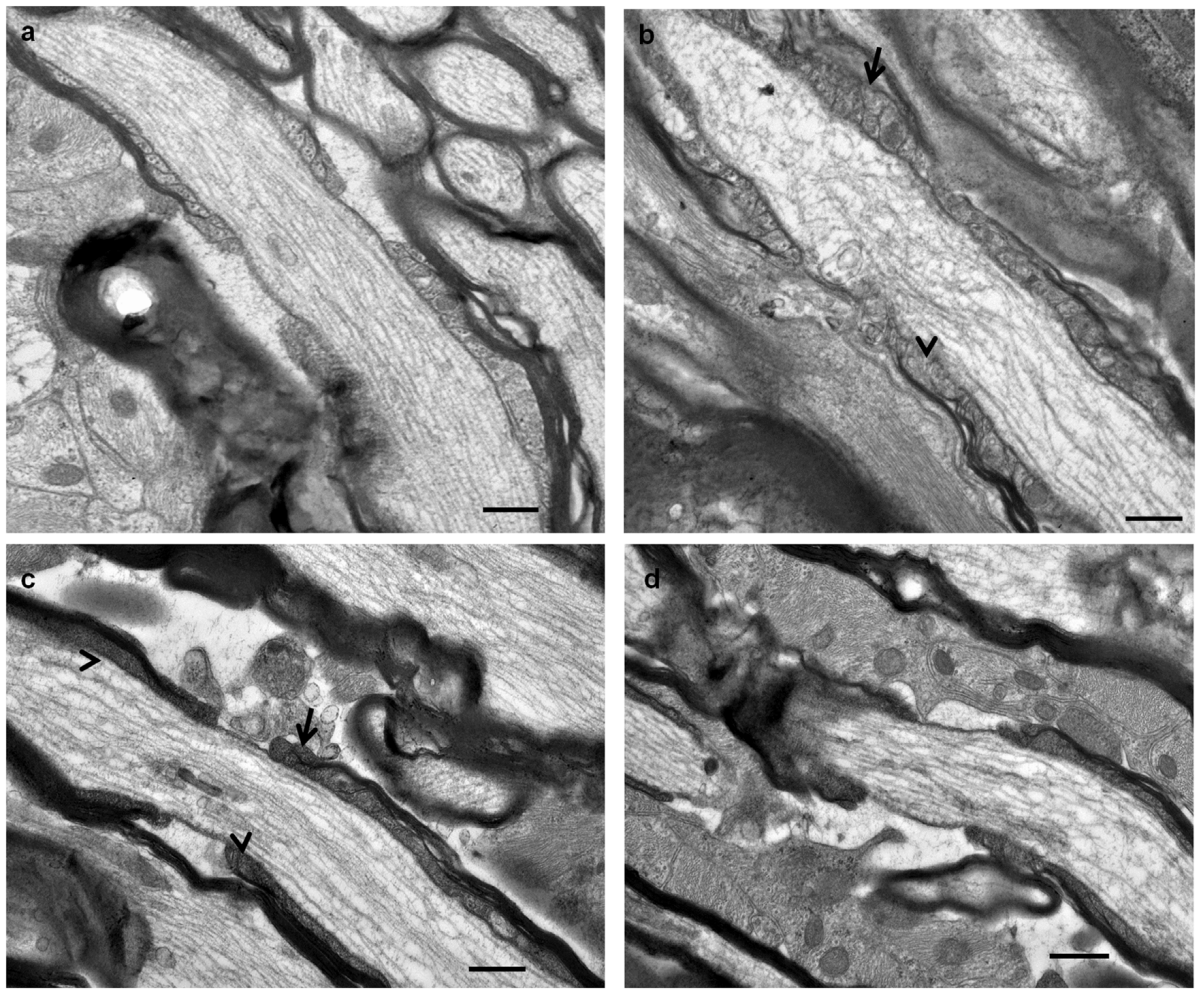
Representative TEM images from normal ventral ON (a) and from day 1 after injury (b–d). Note the disorganisation, lack of definition (arrow head) and multi – layering (arrows) in the paranodal loops from ON vulnerable to secondary degeneration (b, c) and the complete breakdown in structure of one paranode in a node/paranode complex (d), scale bar  = 0.5 μm.

Structural integrity of the node/paranode complex was also directly assessed using TEM, thereby providing information on the contacts of oligodendrocytes to the axon at the axoglial junction. Paranodes in normal ventral ON were characterised by defined paranodal loops in compact array (Fig. 3a). In contrast, in ventral ON at 1 day after injury, there was a pronounced disorganisation of the paranodal structure with many paranodal loops ill – defined and/or multilayered (Fig. 3b, c). Furthermore, in support of our immunohistochemical detection of hemi – nodes and single paranodes in ON vulnerable to secondary degeneration, we observed instances where one paranode was highly disrupted (Fig. 3d), likely reflecting a loss of immunoreactivity and associated functional disturbance.

### 670 nm light treatment increases cytochrome c oxidase activity in vivo

**Figure 4:**
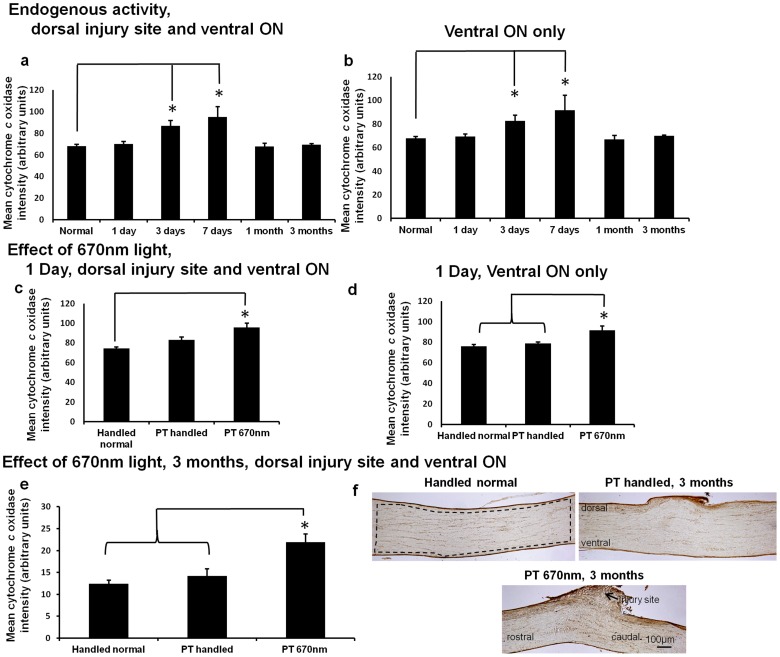
Cytochrome*c* oxidase activity after injury, +/− 670 nm light. Semi – quantification of mean ± SEM cytochrome *c* oxidase activity in ON including both the dorsal injury site and the ventral region vulnerable to secondary degeneration (a) or in ventral ON only (b) from normal animals and 1, 3, 7 days, 1 and 3 months after injury. Similarly, mean ± SEM cytochrome *c* oxidase activity in handled normal, PT handled or PT 670 nm treated animals, encompassing the dorsal injury site and ventral ON (c), or in ventral ON only, 1 day after injury (d). Mean ± SEM cytochrome *c* oxidase activity encompassing the dorsal injury site and ventral ON (defined by region enclosed in dotted lines in F) in PT handled or PT 670 nm treated ON compared to handled normal, 3 months after injury (e); representative images of cytochrome *c* oxidase activity histochemistry at 3 months (f), scale bar  = 100 μm (n = 4–5 animals/group), * significant differences indicated (p≤0.05), PT is partial ON transection injury.

Given that increased CML immunoreactivity in oligodendrocytes is associated with altered node/paranode structure (Figs. 1–3), we asked whether an antioxidant strategy would limit these myelin – related changes. Cytochrome *c* oxidase is a mitochondrial enzyme and a photoacceptor of light at 670 nm; treatment with 670 nm light has been shown to increase the activity of the enzyme, enhance oxidative metabolism in a range of model systems [23], [39], [40] and reduce an indicator of oxidative stress [28]. We first assessed endogenous cytochrome *c* oxidase activity at increasing times after injury in our model (without 670 nm treatment), using semi – quantitative assessments of histochemical staining intensity in longitudinal sections. We observed increased cytochrome *c* oxidase activity at 3 and 7 days after injury when assessing the ON including both the dorsal injury site and the ventral region (Fig. 4a, p≤0.001, dF  = 5), and also when assessing ventral ON only (Fig. 4b, p = 0.002, dF  = 5).

We next confirmed that 670 nm light treatment of animals increased cytochrome *c* oxidase activity in our model. At 1 day after injury, using the same methods as described above, cytochrome *c* oxidase histochemical activity in longitudinal sections of injured, 670 nm treated ON (PT 670 nm) was significantly increased compared to handled normal animals, when assessing the ON including both the dorsal injury site and the ventral region vulnerable to secondary degeneration (Fig. 4c, p = 0.002, dF  = 2). It was also significantly increased compared to both handled normal and handled injured (PT handled) in ventral ON alone (Fig. 4d, p = 0.010, dF  = 2). When assessed following 3 months of 670 nm light treatment after injury, cytochrome *c* oxidase activity remained significantly increased compared to normal and PT handled ON (ON including both the dorsal injury site and the ventral region, p = 0.004, dF  = 2, Fig. 4e, representative images in Fig. 4f). Note that the dorsal injury site is uppermost in each image and the appearance of increased scar tissue with 670 nm treatment is merely a function of the location of the section within the plane of the nerve.

**Figure 5:**
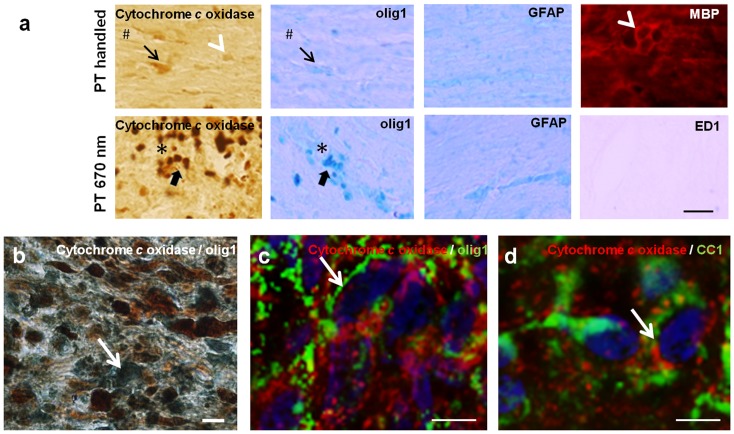
Cytochrome*c* oxidase histochemical staining associated with oligodendrocytes. (a) Representative images of immunohistochemical identification of olig1^+ve^ oligodendrocytes in longitudinal sections of ON, adjacent to sections stained for cytochrome *c* oxidase activity, from PT handled and PT 670 nm treated animals, 1 day after injury. Representative images of MBP, ED1^+ve^ activated microglia/macrophages and GFAP^+ve^ astrocytes are from non – adjacent sections as the cytochrome *c* oxidase staining. Asterixes or hashes indicate common structural feature in adjacent section (putative blood vessel). Arrows or broad arrows indicate similar structural features in adjacent sections, white arrow head indicates a similar structural feature in a non – adjacent section; scale bar  = 20 μm, PT is partial ON transection injury. (b) ON from a PT 670 nm treated animal, 1 day after injury, co-stained for cytochrome *c* oxidase activity (brown) and olig1^+ve^ oligodendrocytes (blue), representative single image along the z axis demonstrating colocalisation (purple cells, example indicated with white arrow), scale bar  = 10 μm. Pronounced cytochrome *c* immunoreactivity (red) in olig1^+ve^ (c) or CC1^+ve^ (d) oligodendrocytes (green) indicated by arrows. Sections were co-stained with Hoechst nuclear stain (blue), assessed in single images within the z plane, scale bar  = 10 μm.

When examined in adjacent sections of ON +/− 670 nm light at 1 day after injury, histochemical staining of cytochrome *c* oxidase activity in ventral ON appeared to colocalise with olig1^+ve^ oligodendrocyte immunoreactivity that also increased with injury (arrows, Fig. 5a). Cytochrome *c* oxidase staining did not resemble the pattern of GFAP^+ve^ astrocyte immunoreactivity in non – adjacent sections (Fig. 5a). Non – adjacent sections were used to compare patterns of immunoreactivity as the limited number of sections directly adjacent to cytochrome *c* oxidase stained sections were used for identification of oligodendrocytes; the focus of the current study. MBP immunoreactivity (note, non – adjacent section) appeared to surround features reminiscent of olig1^+ve^ oligodendrocytes (white arrow heads, Fig. 5a), rather than directly mirroring cytochrome c oxidase activity. This is as expected given the mitochondrial localisation of the enzyme and the lack of mitochondria in compact myelin internodes. Double fluorescence immuno – labelling with antibodies to MBP and olig1 was not possible due to high background of fluorescence based immunoreactivity following the paraformaldehyde/glutaraldehyde perfusion fixative required for cytochrome *c* oxidase histochemistry; streptavidin based detection of these markers using light microscopy was not convincing due to antigen masking. The cellular material heavily stained for cytochrome *c* oxidase activity in PT 670 nm sections appears to include olig1^+ve^ oligodendrocytes (broad arrows), and did not appear to be ED1^+ve^ activated microglia/macrophages (non – adjacent section devoid of blue ED1^+ve^ cells, Fig. 5a). Co – staining of longitudinal sections for cytochrome *c* oxidase activity and olig1 immunoreactivity, with visualisation of a single image along the z axis, indicated colocalisation (purple cells, Fig. 5b), although the cytochrome *c* oxidase staining somewhat masked olig1 immunoreactivity.

Cytochrome *c* oxidase immunoreactivity (as opposed to activity assessed histochemically) was also assessed at 1 day after injury in ON from animals treated with the 670 nm light. Immunohistochemical analysis of cytochrome *c* oxidase in individual images within the z plane indicated that immunoreactivity of the enzyme was present in cell somata of oligodendrocytes also immunoreactive for olig1^+ve^ or CC1^+ve^ of ventral ON at this time. Note that cytochrome *c* oxidase immunoreactivity was widespread throughout the section, as anticipated for this mitochondrial enzyme. Also as expected, the immunoreactivity of mitochondrial cytochrome *c* oxidase did not precisely colocalise with the CC1 or olig1 antigens, but were present in the same cells as indicated by Hoechst nuclear staining. Cytochrome *c* oxidase immunoreactivity was also not present in myelinating processes, given the mitochondrial location of this enzyme (Fig. 5c, d respectively).

### Effects of short-term 670 nm light treatment on ROS /RNS and CML

**Figure 6:**
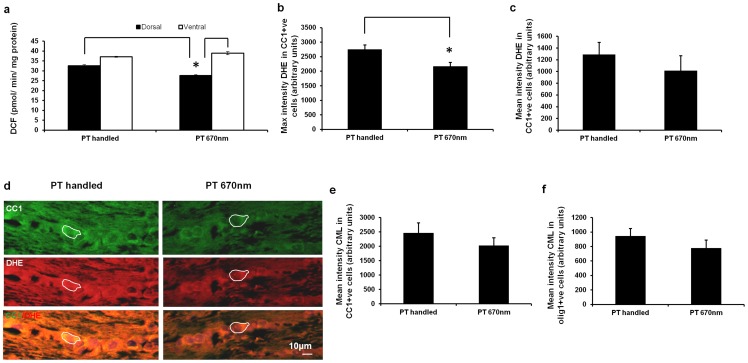
Effects of 670 nm light treatment on oxidative stress indicators. (a) Mean ± SEM ROS/RNS assessed as DCF fluorescence in pooled homogenates from dorsal or ventral ON from PT handled or PT 670 nm treated animals, 1 day after injury (6 animals pooled per time point, assayed in duplicate). Semi – quantification of maximum (b) or mean (c) ± SEM intensity of DHE staining in CC1^+ve^ oligodendrocytes in ventral ON of PT handled or PT 670 nm treated animals, 1 day after injury, assessed by tracing identified cells in single images in the z axis; with (d) representative images, example of identified cells indicated, scale bar  = 10 μm. Similarly, mean ± SEM intensity of CML immunoreactivity in CC1^+ve^ (e) or olig1^+ve^ (f) oligodendrocytes in ventral ON of PT handled or PT 670 nm treated animals, 1 day after injury. PT is partial ON transection injury.

At 1 day after injury, ROS/RNS in dorsal ON homogenates from 670 nm light treated animals was decreased compared to ON of injured handled controls or ventral ON of 670 nm light treated animals (DCF fluorescence assay, Fig. 6a, p = 0.014, dF  = 4). As expected from Fig. 1a, DCF in normal handled ON homogenates was not different to injured handled ON (PT handled) at this time (dorsal or ventral, p>0.05, data not shown). Note that we do not compare DCF values between different experiments (e.g. Fig. 1a and 6a), due to the highly reactive nature of the measured product. There was no significant change in ROS/RNS in ventral or dorsal ON following 7 days of 670 nm light treatment, compared to ON of injured handled controls (data not shown). While our observed reduction in ROS/RNS in ON homogenates following 670 nm light treatment was relatively modest and confined to dorsal ON at day 1, the volatility of reactive species and subsequent oxidation reactions may have prevented detection of more substantial changes.

DHE staining specific to oligodendrocytes of ventral ON was significantly reduced with 670 nm light. Semi – quantitative analysis of single slices within the z plane revealed that the maximum intensity of DHE staining for ROS that colocalised with ventral CC1^+ve^ oligodendrocytes was reduced with 670 nm light treatment at 1 day after injury, compared to injured handled controls (Fig. 6b, p = 0.034 Mann – Whitney test, dF  = 3, representative images Fig. 6d). Mean (as opposed to maximum) intensity of DHE staining in CC1^+ve^ oligodendrocytes tended towards a reduction with 670 nm light, but did not reach significance (Fig. 6c, p = 0.18, dF  = 3); DHE staining in olig1^+ve^ oligodendrocytes was unchanged (data not shown). The significant decrease we observe in the maximum but not the mean intensities of DHE staining following 670 nm light treatment indicates heterogeneity of staining. Our results imply that some cells have a high intensity of DHE staining following injury that is no longer present following 670 nm light treatment, reflected in a significantly lower maximum DHE intensity. It is worth noting that the handling of normal animals required for the 670 nm light treatment, and therefore also for the appropriate handling controls, resulted in a significant increase in DHE staining in ventral CC1^+ve^ cells at day 1 (maximum intensity of normal unhandled  = 2072.8±204.0; normal handled  = 3244.7±282.0, p = 0.014, dF  = 3, mean intensity of normal unhandled  = 869.1±91.6; normal handled  = 1653.9±162.0, p = 0.014 dF  = 3, Mann – Whitney tests), likely due to glucocorticoid release [41], [42]. As such, comparisons of DHE staining were confined to injured animals ±670 nm light treatment or handling control. Note that 670 nm light treatment (handled animals) did not reduce superoxide to levels below those in normal unhandled animals (Fig. 6b, c and data above).

CML immunoreactivity that colocalised with CC1^+ve^ oligodendrocytes, assessed in a single image in the z plane of ventral ON, tended towards a reduction with 670 nm light treatment, compared to PT handled control animals, but the reduction was not significant (Fig. 6e, p = 0.58, dF  = 3). Similarly, there was a trend towards reduced CML immunoreactivity in olig1^+ve^ oligodendrocytes of ventral ON from injured animals at 1 day after injury with 670 nm light treatment, compared to PT handled control animals, but the decrease was not significant (Fig. 6f, p = 0.59, dF  = 3). There was no detectable change in ventral myelin lipid, as indicated by fluoromyelin staining, following 670 nm light treatment at 1 day after injury, although it is important to note that there was also no change with injury compared to handled normal animals at this early time after injury (data not shown, p>0.05).

### Short-term 670 nm light treatment reduces abnormalities in paranodes

**Figure 7:**
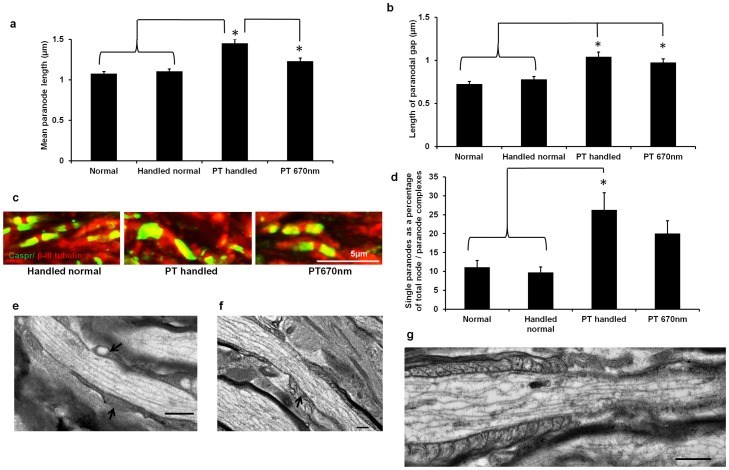
Effects of 670 nm light on node/paranode complexes of ventral ON. Mean ± SEM paranode length (a) and length of the paranodal gap (b) in ventral ON of 670 nm treated and control animals, 1 day after injury; representative images (c). Mean ± SEM percentage of single paranodes in the same groups (d); Caspr^+ve^ paranodes are green, β-III tubulin^+ve^ paranodal gaps are red, scale bar  = 5 μm, * significant differences indicated (p≤0.05), PT is partial ON transection injury. Representative TEM images of PT injured (e) and PT 670 nm light treated (f) node/paranode complexes in ventral ON 1 day after injury. Note the increased definition of the paranodal loops in 670 nm light treated animals, but continued disorganisation (arrows). Representative example of a putative hemi – node in ventral ON from 670 nm light treated animal, with one half of the node/paranode complex clearly defined and the other disorganised (g), scale bars for TEM images  = 0.5 μm.

670 nm light treatment reduced paranode lengths in injured ventral ON at 1 day (p≤0.001, dF  = 3), to levels not different from normal (p>0.05, Fig. 7a, c); the lengths of the paranodal gap (as an indication of node length) were unaffected by 670 nm light (Fig. 7b). Furthermore, the significant increase in the percentage of single paranodes following partial ON transection (p = 0.003, dF  = 3) was not observed when injured animals were treated with 670 nm light (p>0.05, not significantly different from normal, Fig. 7d). However, treatment with 670 nm light did not alter the total percentages of atypical node/paranode complexes, compared to untreated animals (p>0.05, data not shown), indicating continuing hemi – nodes following treatment.

The structure of the node/paranode complex following 670 nm light treatment was also assessed using TEM. It was shown that the disorganisation and lack of definition in the appearance of paranodal loops observed in ventral ON at 1 day after injury (Fig. 7e) was less pronounced when animals were treated with 670 nm light (Fig. 7f). While paranodal loops in the 670 nm treated animals appeared more defined, nevertheless there was still unevenness of distribution and some disorganisation of the paranode, reflecting the incomplete rescue of the node/paranode complex observed in the immunohistochemical analyses. Furthermore there was continued appearance of node/paranode complexes with one half clearly defined and the other disorganised despite a relatively normal appearing node (Fig. 7g). This profound disorganisation in half of the node/paranode complex would likely reduce immunoreactivity of Caspr in the paranode and explain the continuing presence of hemi – nodes in immunohistochemical analyses.

### Long– term 670 nm light treatment is neuroprotective and improves function

**Figure 8:**
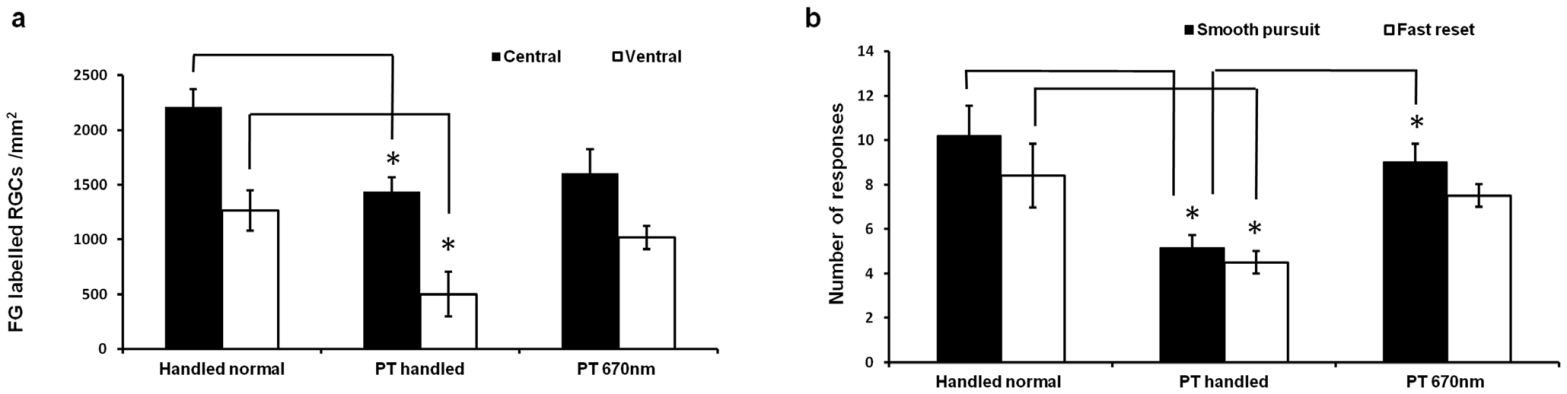
Effects of 670 nm light on RGC numbers and visual function. Mean ± SEM retrograde labelled RGC numbers (central or ventral retinal regions) (a) and responses in the optokinetic nystagmus test of visual function (smooth pursuits or fast resets) (b), in 670 nm treated or control animals 3 months after injury, * significant differences indicated (p≤0.05) (n = 4–5 animals / group), PT is partial ON transection injury.

It is important to know whether prevention of early paranode abnormalities and sustained long – term 670 nm treatments are associated with neuroprotection and preservation of function. As expected, there was a significant decrease in numbers of retrogradely labelled RGCs in central and ventral retina following injury (PT handled compared to handled normal, ventral retina, p = 0.012, dF  = 2, Fig. 8a). However, there was no significant loss of RGCs in central or ventral retinae following injury when animals were treated with the 670 nm light (PT 670 nm compared to handled normal, p>0.05). RGCs in central retinae are vulnerable to death from both the primary injury and from secondary degeneration, whereas RGCs in ventral retinae are vulnerable to death from exclusively secondary mechanisms [6]. As such, 670 nm light treatment limited the loss of RGCs vulnerable to secondary degeneration, particularly those in ventral retina (Fig. 8a). The lower numbers of retrogradely labelled RGCs in ventral compared to central retina of normal and of injured animals are as described [6].

Importantly, preservation of RGCs was associated with improvements in visual function outcomes. Long – term 670 nm light treatment significantly increased numbers of smooth pursuit responses in the optokinetic nystagmus test of visual reflex, compared to injured control animals (p = 0.0156, dF  = 2, Fig. 8b), and both smooth pursuit and fast reset responses in 670 nm light treated animals were not different to responses by normal handled animals (p>0.05).

## Discussion

We have demonstrated *in vivo* that, in ventral ON specifically vulnerable to secondary degeneration, oligodendrocytes show early signs of oxidative stress, with increases in the advanced glycation end – product CML. Node and paranode structure is also disrupted, with paranode lengthening and disorganisation temporally mirroring oxidative stress changes in oligodendrocytes. We used light delivered by LED, at a wavelength of 670 nm, as a tool to control oxidative stress. We provide data to strengthen the premise that 670 nm light increases cytochrome *c* oxidase activity that appears to colocalise with oligodendrocytes. Short – term 670 nm light treatment is associated with decreases in ROS at the injury site, decreased superoxide in myelinating oligodendrocytes and concurrent reductions in paranode abnormalities in ON vulnerable to secondary degeneration. Long – term treatment with 670 nm light helps to preserve RGCs vulnerable to secondary degeneration and maintains behavioural visual function. Light at 670 nm, delivered by LED array, may serve as a practical therapeutic strategy to limit paranode abnormalities and preserve white matter function.

### Indicators of oxidative stress in oligodendrocytes vulnerable to secondary degeneration

Oxidative stress in white matter tracts affected by traumatic injury has been established [10], . Here we extend these findings to directly demonstrate *in vivo* that an oxidative stress indicator can be specifically detected in oligodendrocytes vulnerable to secondary degeneration. Oxidative stress does not necessarily lead to death of oligodendrocytes, as we see no change in olig2^+ve^ oligodendrocyte numbers (identifying predominantly oligodendrocytes across a range of maturation states [45], [46]) at 28 days after injury [8], yet our observed structural changes in the paranode and reported myelin decompaction at later time points after injury [14] indicate oligodendrocyte dysfunction. Our results are in accordance with reports of altered glutamate receptor and ion channel function as well as oxidative stress in susceptible oligodendrocytes in pre – or peri – natal white matter injury, leading to cerebral palsy, multiple sclerosis and demyelinating disease [3], [17], [47]. We demonstrate that increased CML in oligodendrocytes vulnerable to secondary degeneration is associated with lengthening of the paranode and paranodal gap, accompanied by disorganisation of the paranode at 1 day after injury. Paranodal disorganisation is likely to result in disruption of the axoglial junction and saltatory conduction [19], [20], perhaps leading to later myelin decompaction and contributing to long – term loss of function in our model [14]. Similarly, spinal cord white matter exposed to glutamate exhibited paranodal disruption and node lengthening, with associated conduction deficits [21]. Increased paranode length resolved at 7 days after injury. However, while node length also transiently reduced at this time, it increased again at later time points, perhaps reflecting sustained inflammatory cell infiltration and increasing myelin decompaction in ON vulnerable to secondary degeneration [8], [14]. The reason for the restoration of normal paranode lengths but not node lengths at later times after injury is not yet clear, but may reflect vulnerability of specific ion channels to redistribution.

Later increases in DCF fluorescence initially appear in contrast to decreased DHE staining in CC1^+ve^ and olig1^+ve^ oligodendrocytes at this time and suggest either that ROS/RNS other than those detected by DHE are responsible for the increase in DCF observed, or that ROS/RNS including superoxide are increased in other cells of the ON. The decrease in DHE staining following injury appeared relatively general across ON sections, suggesting the former explanation is more likely. Assuming DHE is detecting the ROS superoxide in our system, our reported observation of increased immunoreactivity of MnSOD and decreased catalase after partial ON transection may account for DHE decreases [9], [10]. However, we find no change in MnSOD in oligodendrocytes. This finding is in keeping with the neuroprotective role of astrocytes [48], implying oligodendrocyte to astrocyte superoxide transport *via* gap junctions [49], or extracellular release [50]. Lack of increased ROS/RNS at early time points may be due to sensitivity limitations of the assay given small available tissue volume, or rapid turnover of reactive species [37].

### Effects of 670 nm light

Upregulation of cytochrome *c* oxidase activity as a consequence of red/near – infrared light exposure [22], [23] is thought to result in improvements in oxidative metabolism, and our demonstration of reduced reactive species following 670 nm light treatment *in vivo* further supports this. Mechanisms may include increased flux of electrons through the electron transport chain with maintenance of mitochondrial membrane potential, reduced passage through the reverse electron transport chain, nitric oxide release, altered cAMP and increased ATP synthesis, all of which may result in reduced leakage of free radical intermediates [40], [51]. However, studies linking various facets of oxidative metabolism to ROS/RNS production have been contradictory, largely conducted *in vitro* and highly dependent on the conditions employed [4], [52]; as such, direct deductions concerning the mechanism of effects of 670 nm light are difficult. Nevertheless, light at 670 nm improved indices of mitochondrial function following damage to the CNS in a range of model systems, and improved function [26], [40]. Furthermore, red/near – infrared light exposure has been shown to increase antioxidant protective factors including superoxide dismutase and catalase [26], [40], [53]. We have already reported that 670 nm light treatment is associated with reductions in MnSOD in ventral ON astrocytes [28], and this could be expected to be associated with increased DHE staining if superoxide is being detected, but the opposite is observed. Negative feedback loops are likely to be operating under these circumstances, with decreased reactive species as a consequence of 670 nm light treatment (relative to handled controls), potentially leading to reduced MnSOD activation [54].

Cytochrome *c* oxidase activity is not increased in untreated ON vulnerable to secondary degeneration at 1 day after injury, and we see increased oxidative stress in mature oligodendrocytes and significant node and paranode disruptions at this time. However, when cytochrome *c* oxidase activity is increased in oligodendrocytes as a consequence of 670 nm light, reported by us and others [39], we see reduced superoxide in these cells and paranodes are no longer elongated. Furthermore, the contact of myelinating oligodendrocytes at the paranodal junction is more defined. While associative at this stage, our results imply a relationship between reduced superoxide and perhaps oxidative stress in oligodendrocytes, and preservation of the axoglial junction. The lack of an effect of 670 nm light on length of the paranodal gap, indicative of length of the node of Ranvier, implies that additional mechanisms are important in preserving ion channel clustering at the node, and further investigation is required to elucidate mechanisms. Recent reports suggest that myelinating oligodendrocytes are a major source of lactate for axons [55], but rely predominantly on glycolysis for their own energy needs [56]. Cytochrome *c* oxidase mutants did not exhibit demyelination or axonal degeneration [57]. It is possible that the process of injury and secondary degeneration leads to a ‘switch’ to energy metabolism reminiscent of changes that occur at the onset of myelination [57], as from the present study we can conclude that in white matter vulnerable to secondary degeneration, increased cytochrome *c* oxidase activity in oligodendrocytes is associated with improvements in oligodendrocyte associations with the axon.

The increased cytochrome *c* oxidase activity we observed with 670 nm light treatment is likely due to alterations in the redox state of the enzyme, as previously described [22], although increased protein synthesis cannot be excluded. Tissue penetration of light at these wavelengths, including through bone, has been demonstrated by us and others [28], [40], [58]. As such, increases in cytochrome *c* oxidase activity are likely to be due to direct effects on the nerve, but may also impact on retinal somata [59]. While defining the mechanism/ s of RGC preservation by 670 nm light was beyond the scope of the present study, the interdependence of myelin integrity and axonal structure [49], [55] indicate that the observed maintenance of the paranode may contribute to RGC preservation. Importantly, regardless of mechanism, visual function is maintained in animals treated for 3 months with the 670 nm light. Improvements in vision are likely to be a composite of protection of neurons vulnerable to secondary degeneration and early preservation of the axoglial junction ensuring continued saltatory conduction. In conclusion, light therapy with 670 nm LED may provide a useful strategy for preserving both neurons and the axoglial junction following partial injury to the adult CNS.

## Materials and Methods

### Animals and surgery

Female adult PVG hooded rats (160–190 g, ∼3 months) were bred at the Animal Resources Centre (Murdoch, WA) and maintained in standard housing with food and water *ad libitum* in a 12 hour light/dark cycle. “Principles of laboratory care” (NIH Publication No. 86–23) were followed and this study was approved by The University of Western Australia's Animal Ethics Committee. Rats were anaesthetised intraperitoneally (i.p.) with xylazine (llium xylazil, Troy Laboratories, 10 mg kg^−1^) in combination with ketamine (Ketamil, Troy Laboratories, 50 mg kg^−1^) and euthanased with Euthal (Pentobarbitone sodium 850 mg kg^−1^, Phenytoin sodium 125 mg kg^−1^, i.p.). Partial transection (PT) of the ON was performed as described previously [6], [8]. Briefly, skin overlying the skull was incised along the midline, retracted and the right ON accessed by gently deflecting the Harderian lachrymal gland immediately behind the right eye. The nerve parenchyma was exposed approximately 1 mm behind the eye by making a slit in the *dura mater* with ophthalmic scissors. A controlled 200 μm cut (approximately one quarter of ON width) was made in the dorsal aspect of the ON using a diamond radial keratotomy knife (Geuder), the depth of the incision determined by the protrusion of the blade beyond a surrounding guard. Care was taken not to stretch the ON or damage major ophthalmic blood vessels. The skin was sutured and animals recovered on a warming blanket. Rats were treated with analgesic (Carprieve, 2.8 mg kg^−1^; subcutaneously). No post – operative infections were observed. For immunohistochemical and oxidative stress indicator assessments not involving treatment with 670 nm light, animals were euthanased at 1, 3, 7 days, 1 month or 3 months (n = 6/group/outcome measure) following partial ON transection and results were compared to normal, uninjured animals. Sham operated animals, in which the ON is surgically accessed and the dura opened but the ON not cut, have been shown to be not different from normal in a range of outcome measures including retinal ganglion cell (RGC) numbers, MnSOD immunoreactivity and visual function [8].

### 670 nm light treatment and assessment of visual function

670 nm LED light treatment was administered using a VET 75 LED array (Quantum Devices) positioned 3 cm over the dorsal surface of the entire head of the animal. The device delivers light with a centre wavelength of 670 nm and a full – width at half maximum bandwidth of approximately 22 nm emitting 5 joules of energy per 88– second dose at 60 mW/cm^2^. Additionally the device is air cooled. Choice of treatment duration and administration using the VET 75 LED array was based on previous *in vivo* studies demonstrating beneficial effects [28]. Specifically, lower doses shown to be effective in studies assessing treatment of retinal damage with 670 nm light [25] were not effective at preserving visual function following partial ON transection [28], likely due to increased penetration requirements. 670 nm light treatment groups received a treatment immediately following the transection surgery, under the influence of anaesthesia. Thereafter, treatment was without anaesthesia, for durations of 30 min/day, delivered while animals were loosely held by an investigator such that the animal's head was 3 cm below the light. Animals showed minimal signs of stress and quickly became accustomed to the treatment. Core body temperatures were monitored by rectal thermometer and shown not to increase by more than 1°C.

Short – term effects of 670 nm light on reactive oxygen species/reactive nitrogen species (ROS/RNS) production, immunohistochemical outcomes or cytochrome *c* oxidase activity, (n = 5–6/group/outcome measure) were assessed at day 1 after injury as this was the early time point with the most pronounced effects on oxidative stress and node/paranode structure. Animals were treated immediately after surgery while still under anaesthesia and at 1 day following injury (i.e. a total of two treatments). Two control groups were used: normal handling controls, where normal animals received anaesthetic only (handled normal), and partial transection handling controls, where animals received partial ON transections under anaesthesia (PT handled). Both of these control groups were held for 30 min, 1 day following surgery, in the same way as the 670 nm light treatment group; however they were not exposed to 670 nm light. Animals were euthanased at 1 day following partial ON transection immediately following treatment/handling. An additional control group that was not injured, anaesthetised or handled was also included for some experiments (normal).

To determine long term effects of 670 nm light on RGC numbers and visual function, animals (n = 4–5/group) were treated immediately following the transection under anaesthesia and thereafter, 30 min a day, 6 days/week for three months, without anaesthesia as described above. The final treatment was conducted on the day prior to perfusion. The animals were treated while anaesthetised on the days of surgery for both temporary suture of eyelids on left, non-operated eyes prior to behavioural assessments of visual function and for subsequent removal of sutures and retrograde administration of fluorogold (FG, Fluorochrome). It was necessary to temporarily suture shut the left non-operated eye so that all responses were due to visual function in the injured right eye +/−670 nm light treatment. Similar control groups were used as described above, but the normal control that was not injured, anaesthetised or handled was omitted. Visual function was evaluated by examining the optokinetic nystagmus visual reflex in the injured right eye as described [8] and ONs were histochemically assessed for cytochrome c oxidase activity.

### Assessment of RGC numbers

Intact RGCs with axons projecting to the superior colliculus (SC) were labelled with FG to assess RGC numbers following injury and 670 nm light treatment at 3 months, with experimental groups as described above. FG was applied 3 days before sacrifice by aspirating overlying cortical tissue and applying 5 µL of 5% FG dissolved in 10% DMSO in a gelfoam pledget to the dorso – lateral surface of the SC, where it joins the optic tracts [8], [60], [61]. Following perfusion and fixation, retinae were mounted whole onto Superfrost plus slides (Menzel-Gläser) (ganglion cell layer uppermost), briefly air dried and coverslipped with ProLong Gold® anti – fade reagent (Invitrogen). FG positive RGCs were visualised using UV fluorescence on a Leitz Diaplan microscope, by two independent observers blinded to the treatment identity of the animals. Six randomly selected fields of view (250 µm×125 µm) were assessed for each animal, 3 each in central and ventral retinae and results expressed as RGCs/mm^2^ in each retinal region. Following partial ON transection RGCs in central retinae are vulnerable to both the primary injury and secondary degenerative events, whereas RGCs in ventral retinae succumb exclusively to secondary degeneration [6].

### Dichlorodihydrofluorescein fluorescence assessments of ROS/RNS

Segments of ON of approximately 2 mm in length surrounding the injury site, or equivalent location in normal ON, were excised for ROS/RNS assessments while animals were under anaesthesia. ON segments were assessed whole or dissected into dorsal and ventral halves on a microscope slide sitting on a metal plate overlying dry ice to ensure tissue remained frozen, then rapidly deep frozen in liquid nitrogen before storage at −80°C. Dorsal ON is affected by the primary injury and ventral ON is vulnerable to secondary degeneration [6]. Tissue segments (6/group) were pooled and homogenised in phosphate buffered saline (PBS) using a glass Dounce hand – held homogeniser (Wheaton) on ice, centrifuged at 10,000 g for 5 min and the supernatant stored at −80°C. Duplicate samples were assayed for ROS/RNS using a kit detecting dichlorodihydrofluorescein (DCF) fluorescence (Oxiselect ROS/RNS, Cell Biolabs) as per manufacturer's instructions, and expressed relative to protein concentration of pelleted material, as determined using a micro BCA protein assay kit (Pierce).

### Immunohistochemistry and dihydroethidium staining

Animals were transcardially perfused with 0.9% NaCl followed by 4% paraformaldehyde in 0.1M phosphate buffer, pH = 7.2 (n = 5–6/group). ONs were dissected and postfixed in 4% paraformaldehyde for 24 hours at 4°C. The tissue was cryoprotected in 15% sucrose in PBS overnight and then frozen in optical cutting temperature (OCT) compound. Longitudinal optic nerve sections (14 µm thick) were collected on Superfrost Plus slides (Menzel-Gläser) and stored at −80°C. Slides were air dried for an hour, washed in PBS (pH 7.2−7.4) for 5 min and PBS +0.2% Triton-X 100 for 10 min. Sections were incubated with primary antibodies: mouse monoclonal anti – β-III tubulin to identify RGCs (1∶750, Covance); rabbit anti – Na_v_1.6 to label sodium channels at the node (1∶100, Alamone Laboratories); rabbit polyclonal anti – Caspr to identify paranodes (1∶750, Abcam); mouse monoclonal anti – carboxymethyl lysine, an advanced glycation end – product (CML, 1∶500, CosmoBio); rabbit polyclonal anti – olig1 (1∶500, Abcam) to identify oligodendrocytes and mouse monoclonal anti – CC1 (1∶500, Calbiochem) to identify mature oligodendrocytes; mouse anti – cytochrome c oxidase (MTCO1, 1∶400, Abcam); rabbit anti – astrocyte glial fibrillary acidic protein to identify astrocytes (GFAP; 1∶1000, Dako); rabbit anti – myelin basic protein to label myelin (MBP, 1∶500, Abcam); mouse anti – ED1 to identify activated microglia/macrophages (1∶1000, Millipore) diluted in 5% normal donkey serum (NDS, where required) + PBS +0.2% Triton-X 100 in a humidified chamber overnight at 4°C. After washing with PBS (3×5 min), sections for fluorescence microscopy were incubated with Hoechst nuclear stain (1 μg/ml, Invitrogen) and secondary antibodies: AlexaFluor®488, 555 or 647 donkey anti-rabbit or anti-mouse (1∶400; Invitrogen), isotype specific where required, diluted in PBS +0.2% Triton-X 100 for 2 hours at room temperature. For light microscopy, sections were incubated sequentially with either anti – mouse or anti – rabbit biotintylated secondary antibodies, streptavidin – alkaline phosphatase and the alkaline phosphatase chromagen (Abcam, according to manufacturer's instructions). Slides for fluorescence microscopy were washed with PBS (3×5 min) and mounted with ProLong® Gold; slides for light microscopy were dehydrated through graded ethanol and xylene and mounted with entellan (Merck). Dihydroethidium (DHE) staining to detect ROS was conducted after immunohistochemical staining for CC1 or olig1, by incubating the sections in 1 μM DHE in PBS for 40 min at room temperature, protected from light. DHE is thought to interact with superoxide to generate fluorescent 2-hydroxyethidium [37]. While labelling is usually conducted *in vivo* or on live samples *in vitro* [62], changes in staining for reactive species in fixed tissue samples have been found to be not different from those detected in unfixed tissue [63]. H_2_O_2_ is produced predominantly from superoxide and continues to be generated in paraformaldehyde fixed tissue [64], indicating that if present, superoxide will be available to react with added DHE in our fixed optic nerve sections. DHE labelling post-fixation has been used to detect increases in superoxide in acute pancreatitis [65].

### Immunohistochemical and DHE staining: image collection and analysis

Longitudinal ON sections were visualised and photographed using a Nikon Eclipse Ti inverted microscope (Nikon Corporation) at emission wavelengths of 420 nm, 590 nm and 515 nm, with a 20X or 40X/1.3 N.A. oil immersion objective. For each animal, a series of optical images at 0.5 µm increments along the z axis were acquired from the middle 6 µm of a single 14 µm thick section of ON at the injury site, sampling a field of view of 217.5×162.5 µm encompassing the ventral half of the ON (vulnerable to secondary degeneration). All images were collected using Nikon Elements AR software and deconvolved using autoquant blind deconvolution. Representative images of node/paranode complexes were taken with a Leica TCS SP2 AOBS Multiphoton Confocal microscope (Leica Microsystems) at emission wavelengths of 519 nm and 565 nm. Images were collected in 0.5 μm optical z-series sections and reconstructed into a single image with Leica imaging software. For imaging of cytochrome *c* oxidase activity and associated cellular immunohistochemistry, images encompassing the ventral half of the ON were captured using an Olympus Bx50 upright microscope for non-fluorescent sections and a Leitz Laborlux S upright microscope for fluorescent sections.

#### Analyses of node/paranode complexes

Typical node/paranode complexes were characterised by a β-III tubulin^+ve^ area, also immunopositive for Na_v_1.6 and likely to represent the node of Ranvier, flanked by two Caspr^+ve^ clusters (paranodes). The distance between Caspr^+ve^ paranodes (the paranodal gap, presumed to represent the length of nodes [38]), mean lengths of paranodes in typical node/paranode complexes, as well as the widths of paranodes, were measured using collapsed z stacks. For each animal, 30 typical node/paranode complexes were assessed. To examine the relationship between the lengths of the nodes and paranodes, the proportions of the node length (paranodal gap) to the average of the flanking paranodes length (length node/average (paranode 1+ paranode 2) were calculated. The numbers of typical and atypical node/paranode complexes in the central 125×125 µm area of the fields of view were also quantified. Atypical node/paranode complexes were classified as either hemi – nodes or single paranodes. Hemi – nodes were characterised by a β-III tubulin^+ve^ area flanked by only one paranode, with reference to several adjacent slices in the z axis to ensure the other paranode was truly absent. Similarly, single paranodes were characterised by a Caspr^+ve^ cluster not associated with β-III tubulin immunostaining, presumably due to disruption to the axonal structure leading to reduced β-III tubulin immunoreactivity. Results were expressed as percentages of complexes quantified. Orthogonal z-projections of the entire z stack (6 μm) were used to determine that the depth of typical node/paranode complexes did not generally exceed more than 3 individual optical images in the z plane. As such, the majority of atypical complexes were present well within the z stacks and the probability that parts of complexes were missing adjacent to the edge of z-stacks was constant across each of the sections assessed.

#### Analyses of immunointensities and staining intensities

Each image stack was opened in NIH ImageJ software using the ImageJ ND2 reader plugin and each image along the z axis was converted to a TIFF file. For analyses where colocalisation of 2 markers was assessed, a single image in the z plane with the strongest staining at all wavelengths was used to ensure optimal visualisation and true colocalisation. The intensity of immunoreactivity of oxidative stress indicators (CML, DHE) in individual cell types (CC1^+ve^, olig1^+ve^) was determined by tracing around identified oligodendrocyte lineage cells using the polygon tool and collecting intensity measurements from the same area on the DHE or CML image, with outcome measures of maximum or mean intensity. Note that images within experiments were captured at constant exposures and in a single session, but values obtained cannot be compared to values obtained from different experiments where image exposure times would be different.

### Transmission electron microscopy (TEM) analyses

A separate cohort of animals was used to assess node/paranode structure in longitudinal sections using TEM (normal, PT 1 day after injury, PT 1 day after injury and 670 nm treated, n = 3/group). Following perfusion, tissue was processed and sectioned as described [13], [14] using an LKB Bromma, NOVA Ultrotome®. Ultrathin sections (100 nm) encompassing the injury site were mounted on 3.05 mm copper support grids, and post-stained with uranyl acetate and lead citrate for TEM. Ultrathin sections were viewed under a JEOL JEM-2001 (Japan) TEM at an accelerating voltage of 120 kV, at 4000X magnification attached to an Oris SC1000 CCD camera (40008×2672 pixels), which on this microscope corresponded to a field of view size  = 34.2 μm^2^. Approximately 20 node/paranode complexes were imaged per animal.

### Cytochrome c oxidase histochemistry

Animals were transcardially perfused with 2% paraformaldehyde plus 2% glutaraldehyde in 0.1 M phosphate buffer (pH 7.4) containing 4% sucrose (n = 4–6/group). ONs were dissected and postfixed in the same fixative for one hour at 4°C. The tissue was cryoprotected in 15% sucrose in PBS overnight and then cryosectioned as for immunohistochemistry. Cytochrome *c* oxidase histochemistry was conducted by incubating slides for 180 min at 37°C, shaken at a rate of 70 cycles/min, immersed in an incubation media containing 0.055% DAB, 0.022% cytochrome *c* in 0.1 M phosphate buffer (pH 7.4), or by incubating slides for 4.5 hours in the same conditions followed by overnight incubation at room temperature in a similar incubation media, but containing 0.033% cytochrome *c*, as required (note, all slides were treated identically within individual experiments). Slides were rinsed 3×5 min in PBS, dehydrated in stepwise fashion, initially in PBS and for a few min in each of 50%, 70%, 90%, two×100% ethanol, followed by two changes of xylene. Cytochrome *c* oxidase histochemical staining was semi – quantified and reflected the mean intensity of cytochrome *c* oxidase activity. Slides were visualised and imaged using an Olympus Bx50 upright microscope and Olympus DP70 camera. Image intensity of cytochrome *c* oxidase staining in an area extending approximately 175 µm either side of the lesion (or where the injury would be in normal animals) of dorsal plus ventral ON, or ventral ON alone, was analysed using ImageJ analysis software, assessing mean intensity of inverted images and excluding the dura from analysis. The intensity of a standardised area of the background was subtracted from the overall intensity of cytochrome *c* oxidase staining for 3 month 670 nm light outcomes.

### Statistics

Results were analysed using the statistical package StatView for Windows (SAS Institute Inc.). Kolmogorov – Smirnov tests were used to confirm that results were consistent with a normal distribution and Brown Forsythe equality of variance F – tests to assess homogeneity of variances in groups within experiments. One – way ANOVA, followed by Bonferroni/Dunn, Dunnett's or Tukey's *post hoc* tests were used as appropriate (p value from ANOVA given when describing differences between several groups and control, or from *post hoc* test when describing individual comparisons). In some instances, where normal distribution was not assured but variances were equal, Kruskal – Wallis one – way analysis of variance of ranks followed by Mann – Whitney U tests for specific comparisons were used (stated in the text where used, p value from Mann – Whitney U tests and dF from Kruskal – Wallis tests given).
